# Protective Immunity and Safety of a Genetically Modified Influenza Virus Vaccine

**DOI:** 10.1371/journal.pone.0098685

**Published:** 2014-06-13

**Authors:** Rafael Polidoro Alves Barbosa, Ana Paula Carneiro Salgado, Cristiana Couto Garcia, Bruno Galvão Filho, Ana Paula de Faria Gonçalves, Braulio Henrique Freire Lima, Gabriel Augusto Oliveira Lopes, Milene Alvarenga Rachid, Andiara Cristina Cardoso Peixoto, Danilo Bretas de Oliveira, Marco Antônio Ataíde, Carla Aparecida Zirke, Tatiane Marques Cotrim, Érica Azevedo Costa, Gabriel Magno de Freitas Almeida, Remo Castro Russo, Ricardo Tostes Gazzinelli, Alexandre de Magalhães Vieira Machado

**Affiliations:** 1 Laboratório de Imunopatologia, Centro de Pesquisas René Rachou, FIOCRUZ, Belo Horizonte, Minas Gerais, Brasil; 2 Laboratório de Imunoparasitologia, Departamento de Bioquímica e Imunologia, Instituto de Ciências Biológicas, Universidade Federal de Minas Gerais, Belo Horizonte, Minas Gerais, Brasil; 3 Laboratório de Imunofarmacologia, Departamento de Bioquímica e Imunologia, Instituto de Ciências Biológicas, Universidade Federal de Minas Gerais, Belo Horizonte, Minas Gerais, Brasil; 4 Laboratório de Imunologia e Mecânica Pulmonar, Departamento de Fisiologia e Biofísica, Instituto de Ciências Biológicas, Universidade Federal de Minas Gerais, Belo Horizonte, Minas Gerais, Brasil; 5 Departamento de Patologia Geral, Instituto de Ciências Biológicas, Universidade Federal de Minas Gerais, Belo Horizonte, Minas Gerais, Brasil; 6 Laboratório de Vírus, Departamento de Microbiologia, Instituto de Ciências Biológicas, Universidade Federal de Minas Gerais, Belo Horizonte, Minas Gerais, Brasil; 7 Division of Infectious Diseases and Immunology, Department of Medicine, University of Massachusetts Medical School, Worcester, Massachusetts, United States of America; Washington University School of Medicine, United States of America

## Abstract

Recombinant influenza viruses are promising viral platforms to be used as antigen delivery vectors. To this aim, one of the most promising approaches consists of generating recombinant viruses harboring partially truncated neuraminidase (NA) segments. To date, all studies have pointed to safety and usefulness of this viral platform. However, some aspects of the inflammatory and immune responses triggered by those recombinant viruses and their safety to immunocompromised hosts remained to be elucidated. In the present study, we generated a recombinant influenza virus harboring a truncated NA segment (vNA-Δ) and evaluated the innate and inflammatory responses and the safety of this recombinant virus in wild type or knock-out (KO) mice with impaired innate (Myd88 -/-) or acquired (RAG -/-) immune responses. Infection using truncated neuraminidase influenza virus was harmless regarding lung and systemic inflammatory response in wild type mice and was highly attenuated in KO mice. We also demonstrated that vNA-Δ infection does not induce unbalanced cytokine production that strongly contributes to lung damage in infected mice. In addition, the recombinant influenza virus was able to trigger both local and systemic virus-specific humoral and CD8+ T cellular immune responses which protected immunized mice against the challenge with a lethal dose of homologous A/PR8/34 influenza virus. Taken together, our findings suggest and reinforce the safety of using NA deleted influenza viruses as antigen delivery vectors against human or veterinary pathogens.

## Introduction

Influenza A viruses (*Orthomyxoviridae*) have two glycoproteins anchored on the viral envelope: hemagglutinin (HA) and neuraminidase (NA). Hemagglutinin mediates viral entry into the lung epithelial cell by binding the viral particle to cell surface receptors (the sialic acid units), while the neuraminidase cleaves the sialic acid allowing the release of the newly formed viral particles [1].

Recombinant influenza viruses have been proven to be very efficient as antigen delivery vectors [2], [3]. Although some strategies have already been developed to generate recombinant influenza viruses, most of them are hampered by retention of their original virulence [4], [5]. To bypass this, Fuji and colleagues generated recombinant influenza viruses harboring a partially deleted neuraminidase segment, where its catalytic region was replaced by a foreign sequence [6], [7]. Although influenza viruses lacking functional neuraminidase have been found to be highly attenuated in wild type mice, the inflammatory response triggered by those viruses, as well as their safety in immunocompromised hosts remains to be evaluated. [6], [7], [8].

The pro-inflammatory milieu is important for counteracting the viral infection before the development of acquired immunity. It is also responsible for the influenza-induced injury [7], [9], [10], [11]. The unbalanced cytokine and chemokine production by cells from the lung parenchyma is a significant pathological component which plays a major role in amplification of pulmonary damage and collapse in mortality in influenza infected patients [12], [13], [14], [15], [16], [17]. Thus, it is important to improve our knowledge about how recombinant influenza viruses lacking functional neuraminidase modulate the inflammatory immune response in lungs and impact the lung physiology.

Therefore, in the present study, we evaluated the immunopathogenic profile induced by a recombinant influenza virus harboring a truncated neuraminidase segment and its safety for wild type mice and those lacking the innate or the acquired branches of immune response. Our results show that recombinant influenza viruses without functional neuraminidase induce discrete pulmonary inflammatory response and lung damage. In addition, vaccination with this recombinant virus elicits local and systemic acquired specific immune responses which are able to protect mice challenged with homologous highly virulent wild type virus A/PR8/34. Moreover, the recombinant influenza virus harboring a truncated neuraminidase are attenuated even in MyD88 -/- and Rag -/- mice. Overall, our results support the safety of using such genetically engineered influenza vectors carrying heterologous sequences as live bivalent vaccines.

## Results

### Generation and characterization of recombinant viruses

**Figure 1:**
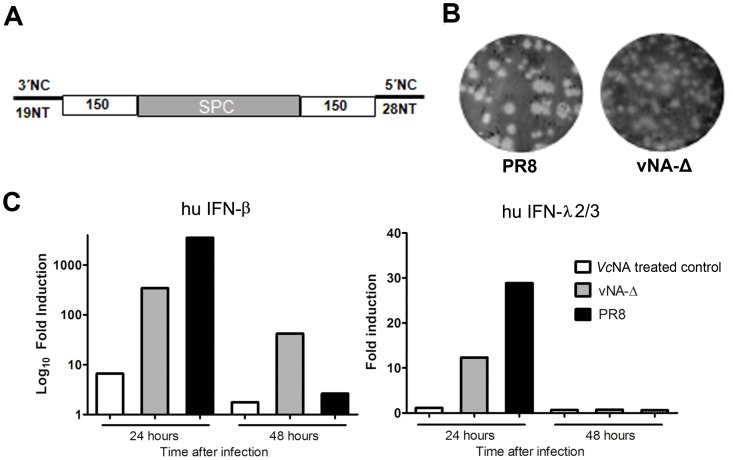
Generation and characterization of recombinant viruses harboring truncated neuraminidase. Deleted neuraminidase segments displaying deletions at 3′ and 5′ extremities were generated as described in Material and Methods. The remaining neuraminidase coding regions are shown in white squares. A spacer sequence was inserted between the 3′ and 5′ moieties (A). Wild type PR8 virus and recombinant vNA-Δ influenza viruses were generated by reverse genetics as described in Material and Methods. The plaque phenotypes of these viruses were assessed by standard agarose plaque assay in MDCK infected cells after 3 days of incubation (B). Confluent monolayers of A549 cells were cultured with DMEM media containing BSA, Trypsin and neuraminidase (*Vc*NA treated control) or infected with wild type PR8 virus or recombinant vNA-Δ at M.O.I of two. The induction of hu-IFN-β (type I, A) and hu-IFN λ2/3 (type III, B) was assessed at different time points by quantitative PCR using lightcycler Real Time PCR Machine (Applied Biosystems; C). Analysis was performed using SDS 2 software. All data are expressed as a ratio relative to *Vc*NA treated control.

Wild type A/PR8/34 virus (herein named PR8) and recombinant influenza (vNA-Δ) harboring a spacer sequence of 660 nucleotides (**figure 1A**) were generated by eight plasmid driven reverse genetics, as described by de Goede [10]. The recombinant vNA-Δ virus displayed lysis plaques on MDCK cells smaller than those of the reverse genetics generated PR8 virus (**figure 1B**) and its infectious titer was 10-fold lower (10^7^ PFU/ml vNA-Δ).

To assert if the generated virus is able to trigger immune response in human epithelial cells we evaluated the induction of type I and III interferons in A549 cells infected with PR8 or recombinant vNA-Δ virus in the presence of exogenous neuraminidase or incubated with the same media without virus (*Vc*NA treated control). At different time points, total cellular RNA was extracted and mRNA levels of human (hu) IFN-β (type I) and hu IFN-λ2/3 (type III) were evaluated by qRT-PCR. The results depicted in **figure 1C** show that both PR8 and recombinant vNA-Δ viruses were able to induce type I and III interferons, which attained their maximal fold induction at 24 hours post-infection.

### Reduced viral loads and attenuated pulmonary inflammatory response in mice inoculated with vNA-Δ virus

**Figure 2:**
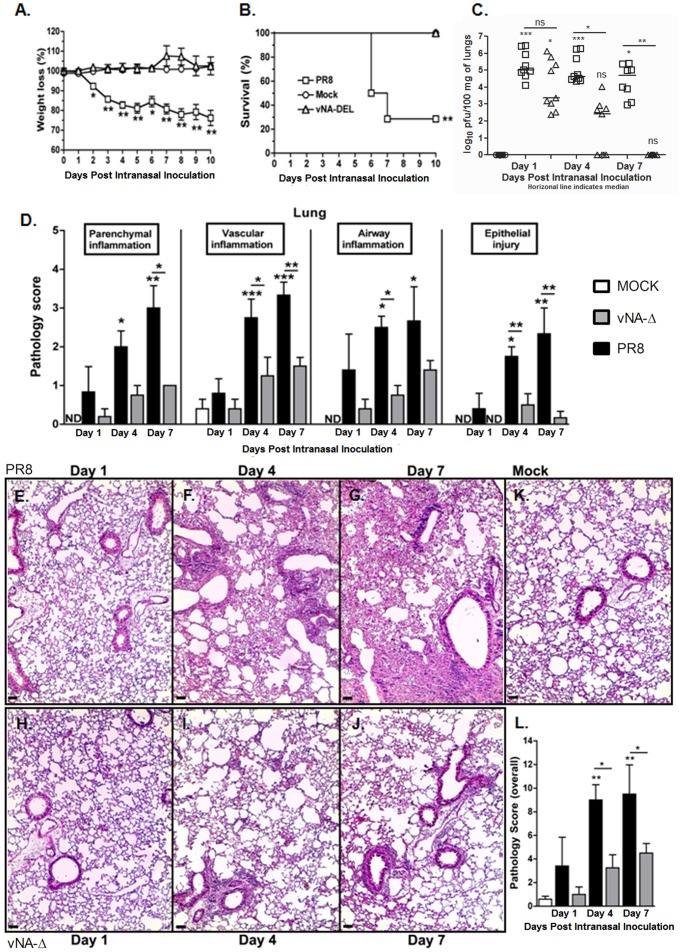
Characterization of virulence and lung inflammation in mice inoculated vNA-Δ. C57BL/6 mice were infected intranasally with 10^5^ PFU Influenza PR8, vNA-Δ or PBS (mock) inoculated (n  =  4–6 in each group). Weight loss (A) and lethality (B) were evaluated over 10 days. Mice were euthanized 1, 4 and 7 dpi and virus titers were quantified in lung (C). The figure shows one representative experiment. Lung pathologic score after infection with influenza PR8 virus or vNA-Δ was assessed in lung slices stained with H&E by a pathologist showing parenchyma, vascular and airway inflammation, and epithelial injury (D). Representative slides of PR8 virus (E, F and G), vNA-Δ (H, I and J) and mock (K) inoculated mice at 1, 4 and 7 dpi. The pathology overall score was determined (L). *n*  =  5 for all groups. Data are presented as mean ± SEM. * and ** for p<0.05 and p<0.01, respectively, when compared to mock or indicated groups (one-way ANOVA, Newman-Keuls).

Mice were anesthetized and inoculated with 10^5^ PFU of PR8 (corresponding to approximately 20 LD_50_) or 10^5^ PFU of vNA-Δ virus. As depicted in **figure 2A**, the animals inoculated with PR8 lose around 25% of body weight and approximately 75% of animals died by 7 days post inoculation (dpi). By contrast, the animals inoculated with 10^5^ PFU of vNA-Δ showed neither weight loss nor death (**figure 2A and 2B**), corroborating the results obtained in previous studies [6], [10]. In addition, animals inoculated with PR8 virus displayed detectable virus in lungs 1, 4 and 7 dpi, whereas the viral titers in lungs of mice inoculated with vNA-Δ dropped dramatically at day 4 and became virtually undetectable 7 dpi (**Figure 2C**).

It is well established that inflammatory response plays a pivotal role in immunopathology during influenza infection [7]. To evaluate the inflammatory response triggered by recombinant vNA-Δ, we inoculated mice with 10^5^ PFU of PR8 virus or recombinant vNA-Δ virus and measured inflammatory parameters at different time points after infection.

Histopathological analysis showed that mice inoculated with wild type PR8 virus displayed macroscopic signs of pneumonia such as petechiae and even hepatization of lungs at 4 and 7 dpi. By contrast, no macroscopic lesions were found in lungs of animals inoculated with vNA-Δ at any time point (data not shown). Moreover, the grading scores demonstrated that inflammatory lesions in lung parenchyma, vessels, airways and epithelial injury in mice inoculated with PR8 virus were significantly higher than those found in animals inoculated with vNA-Δ or inoculated with PBS (mock) at 4 and 7 dpi (**figure 2D**). Importantly, histopathological analysis demonstrated that PR8 inoculation resulted in higher injury due to the inflammatory response (**figure 2E-G**), whereas inoculation with vNA-Δ resulted in only a mild inflammatory response (**figure 2H-J**), similar to PBS inoculated mice (**figure 2K**). Overall, increased inflammatory response in mice inoculated with PR8 virus resulted in a higher pathology score (**figure 2L**), while this parameter was highly reduced in mice inoculated with vNA-Δ.

**Figure 3:**
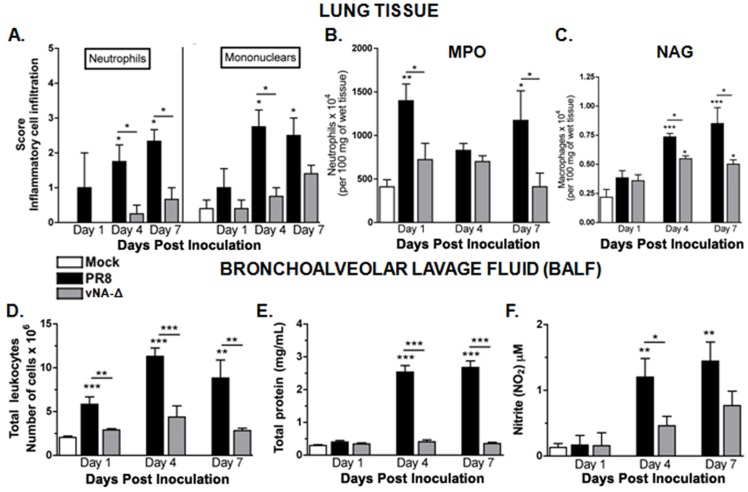
Leukocyte recruitment to the lungs and BAL following vNA-Δ infection. C57BL/6 mice were inoculated with PBS (mock) or infected intranasally with influenza 10^5^ PFU of PR8 virus or vNA-Δ (n = 5). Mice were euthanized and lungs removed 1, 4 and 7 dpi. The recruitment of neutrophils and macrophages/mononuclear (A) cells to the lungs was assessed in lung H&E stained slides. Frozen lungs sections were assessed for Myeloperoxidase (B) and N-acetylglucosaminidase (C) contents, indirect measurements for neutrophils and macrophages, respectively. Mice were euthanized (n  =  6–8 in each group) 1, 4 and 7 dpi and bronchoalveolar lavage was performed. Absolute numbers of airways leukocytes after infection with 10^5^ PFU (D). Total proteins (E) and nitrite (NO2^−^; F) were also determined in BALF. Data are presented as mean ± SEM. *, ** and *** for p<0.05, p<0.01 and p<0.001, respectively, when compared to mock or indicated groups (one-way ANOVA, Newman-Keuls).

Accordingly, histopathological analysis of classical inflammatory parameters showed that there was a high neutrophilic and mononuclear cell infiltrate in the lungs of mice infected with PR8 virus (**figure 3A**). This result was correlated with myeloperoxidase (MPO) activity (**figure 3B**), and with increased levels of N-acetylglucosaminidase (NAG, **figure 3C**), which are markers for lesions mediated by neutrophils and macrophages, respectively. By contrast, those parameters were reduced in mice inoculated with vNA-Δ and comparable to those in PBS inoculated mice (mock). The only exception was the level of NAG, which were increased in the lungs of mice inoculated with vNA-Δ, suggesting an elevation in macrophage accumulation into lung tissue after infection with vNA-Δ (figure 3C).

Consistently, analysis performed on bronchoalveolar lavage (BAL) and fluid (BALF) demonstrated that the overall number of leukocytes, total protein and nitric oxide levels were significantly reduced in vNA-Δ compared with wild-type inoculated mice (**figure 3D-F**). Interestingly, the levels of chemoattractants for neutrophils (KC/CXCL1), monocytes (MCP-1/CCL2), lymphocytes (MIG/CXCL9) and eosinophils (CCL11) were found to be significantly higher in BAL of mice inoculated with PR8, which was correlated with increased cell infiltrate in BAL of animals inoculated with PR8 (**figure S1**). Overall, this data suggests that vNA-Δ inoculation cause mild inflammation in the lung.

### Reduced inflammatory cytokine levels in lungs of mice inoculated with vNA-Δ

**Figure 4:**
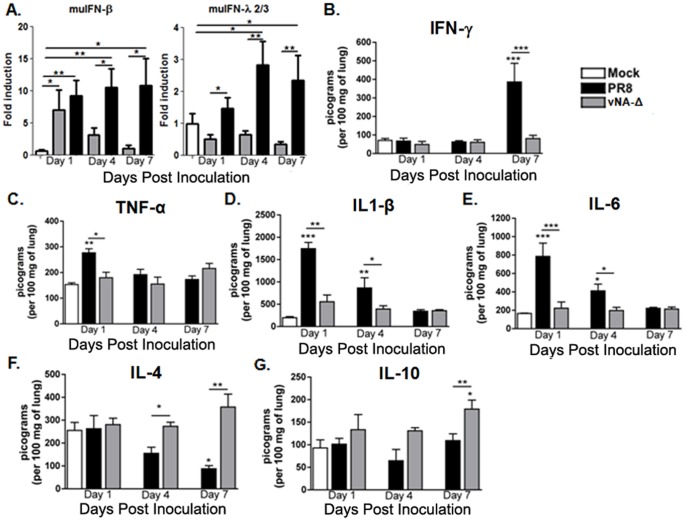
Measurement of cytokines in the lung. C57BL/6 mice were inoculated with PBS (mock) or infected intranasally with 10^5^ PFU influenza PR8 virus or vNA-Δ (n = 5) and euthanized 1, 4 and 7 dpi. The induction of murine IFN-β and IFN-λ2 (A) was measured in lungs by qRT-PCR as described in Material and Methods. The levels of cytokines IFN-γ (B), TNF-α (C), IL-1β (D), IL-6 (E), IL-4 (F) and IL-10 (G) were measured in lung tissue by ELISA. *n*  =  5 for all groups at days 1 and 4, *n*  =  5, 4, 6 for mock, PR8 and vNA-Δ viruses at day 7. Data are presented as mean ± SEM. * ** and *** for p<0.05, p<0.01 and p<0.001, respectively, when compared to mock or indicated groups (one-way ANOVA, Newman-Keuls or unpaired t test (qRT-PCRs).

In order to evaluate if the lack of inflammation could be related to decreased levels of pro-inflammatory cytokines we analyzed the lung tissue of mice inoculated with PR8 or vNA-Δ. By qRT-PCR we detected an increase in muIFN-β and mu IFN-λ2/3 gene expression in the lung of mice inoculated with PR8 virus at all evaluated timepoints. This increase could not be detected in mice inoculated with vNA-Δ virus except by muIFN-β at 1dpi (**figure 4A**). In addition, ELISA performed on lung homogenates showed the production of IFN-γ only in mice inoculated with PR8 (**figure 4B**). Consistently, the pro-inflammatory cytokines TNF-α (**figure 4C**), IL-1β (**figure 4D**) and IL-6 (**figure 4E**) were augmented in mice inoculated with PR8. By contrast, the levels of all measured pro-inflammatory cytokines found in the lungs of mice inoculated with vNA-Δ were similar to those found in PBS (mock) inoculated mice (**figure 4A-E**). Finally, the same cytokine production profile could be observed in BALF of inoculated mice (**figure S2**).

Interestingly, the levels of counter-regulatory cytokines interleukin 4 (IL-4) and IL-10 in lungs of mice infected with PR8 were reduced at day 4 and 7, whereas the levels of those cytokines in the lungs of mice inoculated with the attenuated vNA-Δ were unaltered or slightly increased when compared to PBS (mock) inoculated mice (**figure 4F and G**).

**Figure 5:**
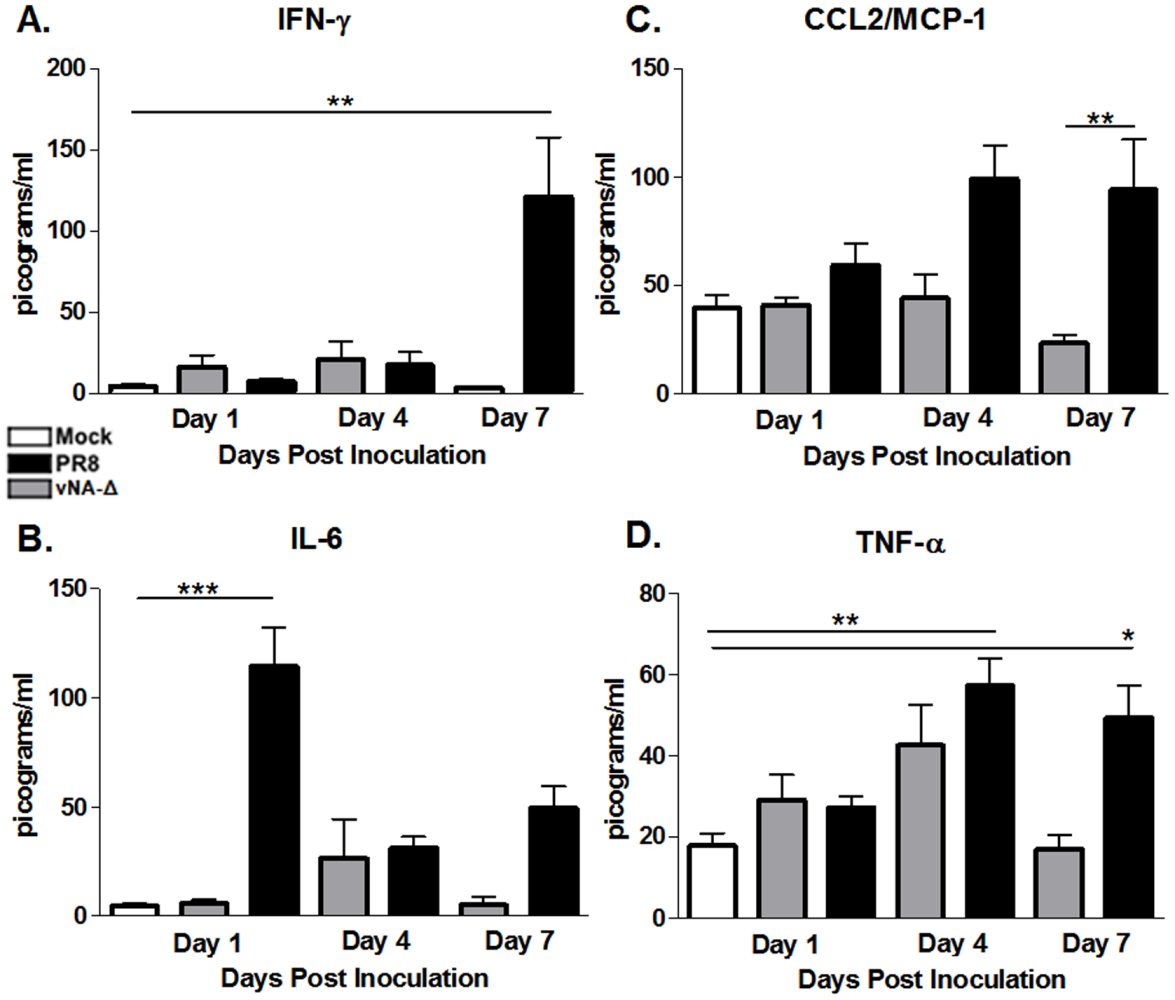
Measurement of cytokines in sera of inoculated mice. C57BL/6 mice were inoculated with PBS (mock; n = 9) or infected intranasally with 10^5^ PFU of influenza PR8 (n = 10-12) or vNA-Δ (n = 9–11) viruses. By CBA, the levels of IFN-γ (A), CCL2/MCP-1 (B), IL-6 (C) and TNF-α (D) were measured in the sera collected from mice at days 1, 4 and 7 after inoculation. Data represents two independent experiments and are presented as mean ± SEM. * ** and *** for p<0.05, p<0.01 and p<0.001, respectively, when compared to mock or indicated groups (one-way ANOVA, Newman-Keuls).

Finally, we assessed the systemic levels of inflammatory or regulatory cytokines in serum collected from inoculated mice at 1, 4 or 7 dpi by Cytometric Bead Array (CBA). No significant cytokine production was detected in sera of mice inoculated with vNA-Δ virus compared to PBS inoculated mice (p>0.05, **Figure 5**). In mice inoculated with PR8 virus, increased levels of IFN-γ and CCL2/MCP-1 were detected at 7 dpi (**figure 5A and 5B**). Increased levels of IL-6 (**figure 5C**) were found only at 1 dpi and TNF-α (**figure 5D**) at days 4 and 7. No significant increase in levels of IL-12p70, and IL-10 were found in serum of animals inoculated with any virus (data not shown). Collectively, these data suggest that PR8 induces a robust local and systemic inflammatory response and reduced levels of counter-regulatory cytokines, which result in increased lung injury when compared to that found in vNA-Δ.

### Evaluation of adaptative immune response elicited by vNA-Δ

**Figure 6:**
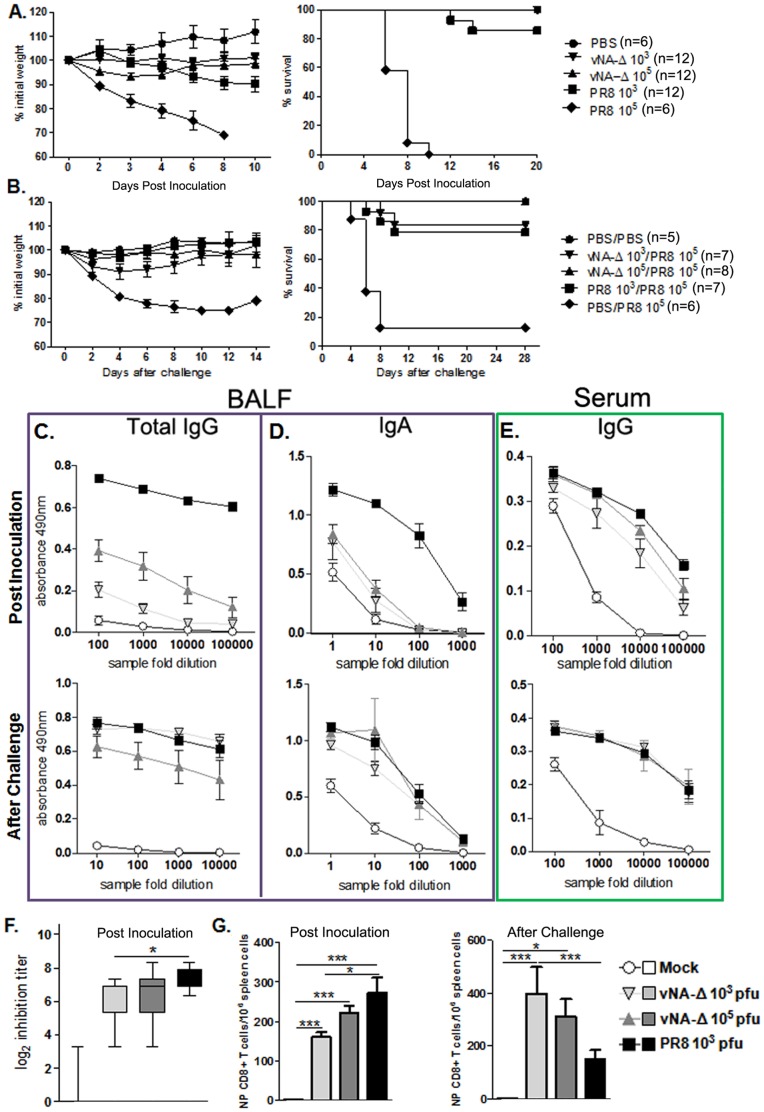
Evaluation of protection and acquired immune response elicited by inoculation with vNA-Δ. C57BL/6 mice were inoculated with PBS (mock) or infected intranasally with 10^5^ PFU Influenza A/PR8 virus, 10^3^ or 10^5^ PFU of vNA-Δ virus. Twenty-one days after the prime-inoculation the animals were challenged with a lethal dose of 10^5^ PFU of PR8. The weight loss and survival were determined after the prime-inoculation (A) and the challenge (B) infection (data represents two independent experiments). Blood and BALF were collected fourteen days after prime-inoculation and challenge infections. Tenfold dilutions of BALF samples were used to determine total IgG (C) and IgA (D) in BALF and total IgG in serum by ELISA (E)(Data depict one representative experiment). Two fold serial dilution of serum was used for the hemagglutinin-inhibition assay (F). n  =  4 for BALF IgA and IgG measures after inoculation. n  =  4, 7, 7, 6 for mock, vNA-Δ 10^3^, vNA-Δ 10^5^, PR8 10^3^ for serum IgG. n  =  4, 5, 6, 5 for mock, vNA-Δ 10^3^, vNA-Δ 10^5^, PR8 10^3^ for hemagglutinin-inhibition assay (Data represents two independent experiments). Spleens of mice (n  =  4) were obtained two weeks after the inoculation or challenge infection. Specific NP CD8+ T cells were assessed by ELISPOT using nucleoprotein (NP) of PR8 ASNENMETM peptide (NP; aa 366–374) as stimulus (G) Data represents two (inoculum) or three (challenge) independent experiments. Data were evaluated by Mann-Whitney test *, ** and *** for p<0.05, p<0.01 and p<0.001 respectively.

Next, we evaluated if the attenuated recombinant influenza virus is able to induce a proper acquired immune response and protect mice against homologous lethal challenge. For this purpose, C57BL/6 mice were anesthetized and inoculated intranasally with PBS (mock inoculation), vNA-Δ or PR8 virus (10^3^ or 10^5^ PFU). Mice inoculated with 10^5^ PFU of PR8 displayed remarkably weight loss and died, whereas most of the animals inoculated with 10^3^ PFU of PR8 survived the inoculation in spite of their weight loss (**figure 6A**). As expected, the animals inoculated with vNA-Δ survived the inoculation without weight change, irrespective of the inoculum used (**figures 6A and 6B** **)**. Three weeks after the first inoculation, mice were challenged with a lethal dose (10^5^ PFU) of PR8 virus. Weight change and mortality was followed over two weeks (**figures 6A and 6B** **)**. As expected, the animals that were previously inoculated with PBS and further challenged with a lethal dose of PR8 displayed abrupt weight loss and high mortality (87%, n = 8). Most of the mice inoculated previously with sub lethal dose of pathogenic PR8 survived the challenge (78%, n = 14). Remarkably, the mice inoculated with 10^3^ PFU of vNA-Δ showed significant weight loss (**figure 6A**) and two of twelve mice died (**figure 6B**). Differently, the animals that were inoculated with 10^5^ PFU of vNA-Δ showed no weight loss (**figure 6A**) and all the animals survived the challenge (**figure 6B**).

Next, we assessed specific antibodies against influenza in Bronchoalveolar Lavage Fluid (BALF) and sera, as well as CD8+ T cells specific for nucleoprotein (NP) in the spleen of mice inoculated with vNA-Δ or PR8, after the prime inoculation and the challenge infection. Measured by ELISA, significantly higher levels of both IgG and IgA were found in the BALF of mice inoculated with PR8 virus (**figure 6C and 6D**). The IgA levels found in mice inoculated with either dose of vNA-Δ were similar. Differently, higher levels of IgG were found in BALF of mice inoculated with 10^5^ PFU of vNA-Δ when compared to those found in BALF of animals inoculated with 10^3^ PFU of vNA-Δ (**figure 6C**). In contrast to BALF, anti-influenza total IgG levels in sera derived from mice inoculated with 10^5^ PFU of vNA-Δ were similar to those found in mice inoculated with PR8, but higher than those inoculated with vNA-Δ 10^3^ (**figure 6E**). Interestingly, the levels of antibodies found in sera (total IgG and IgA) and in BALF (IgA; **figures 6C-E**) of mice inoculated with vNA-Δ were similar to those found in mice inoculated with PR8 after the challenge infection.

Serum hemagglutinin inhibition (HI) titers of PR8 vaccinated mice (log_2_ 7.47±4.85) were similar to those found in sera of mice inoculated with 10^5^ PFU of vNA-Δ (log_2_ 6.92±4.85) and higher than those found in BALF of mice inoculated with 10^3^ PFU of vNA-Δ (log_2_ 6.17±4.17; **figure 6F**). In line with these results, we found that all the animals (n = 5) inoculated with 10^3^ PFU of vNA-Δ harbored virus in the lung after challenge with PR8. By contrast, only one among five animals inoculated with 10^5^ PFU of vNA-Δ had detectable virus in lungs after challenge and the viral load was lower than that found in mice inoculated with PBS and further challenged with PR8 virus (p<0.01, **figure S3**).

Examining the cellular immune response, we found significantly higher number of NP specific CD8+T cells in the spleen of mice inoculated with PR8 virus than those found in mice inoculated with 10^3^ PFU of vNA-Δ after the prime inoculation (**figure 6G**). Differently, two weeks after challenge the number of NP specific CD8+ T cells in spleen of mice inoculated with 10^3^ PFU or 10^5^ PFU of vNA-Δ was significantly higher than those found in spleens of mice inoculated with PR8 virus (**figure 6G**).

Taken together, our results showed that vaccination using recombinant influenza harboring a neuraminidase deficient segment elicits humoral and cell-mediated immune responses. Moreover, the antibody levels, the HI levels and the number of specific CD8+ T cells elicited by inoculation with vNA-Δ are inoculum dependent, resulting in different degrees of protection against the challenge infection.

### Recombinant vNA-Δ is highly attenuated in knock-out mice for innate or acquired branches of immune response

**Figure 7:**
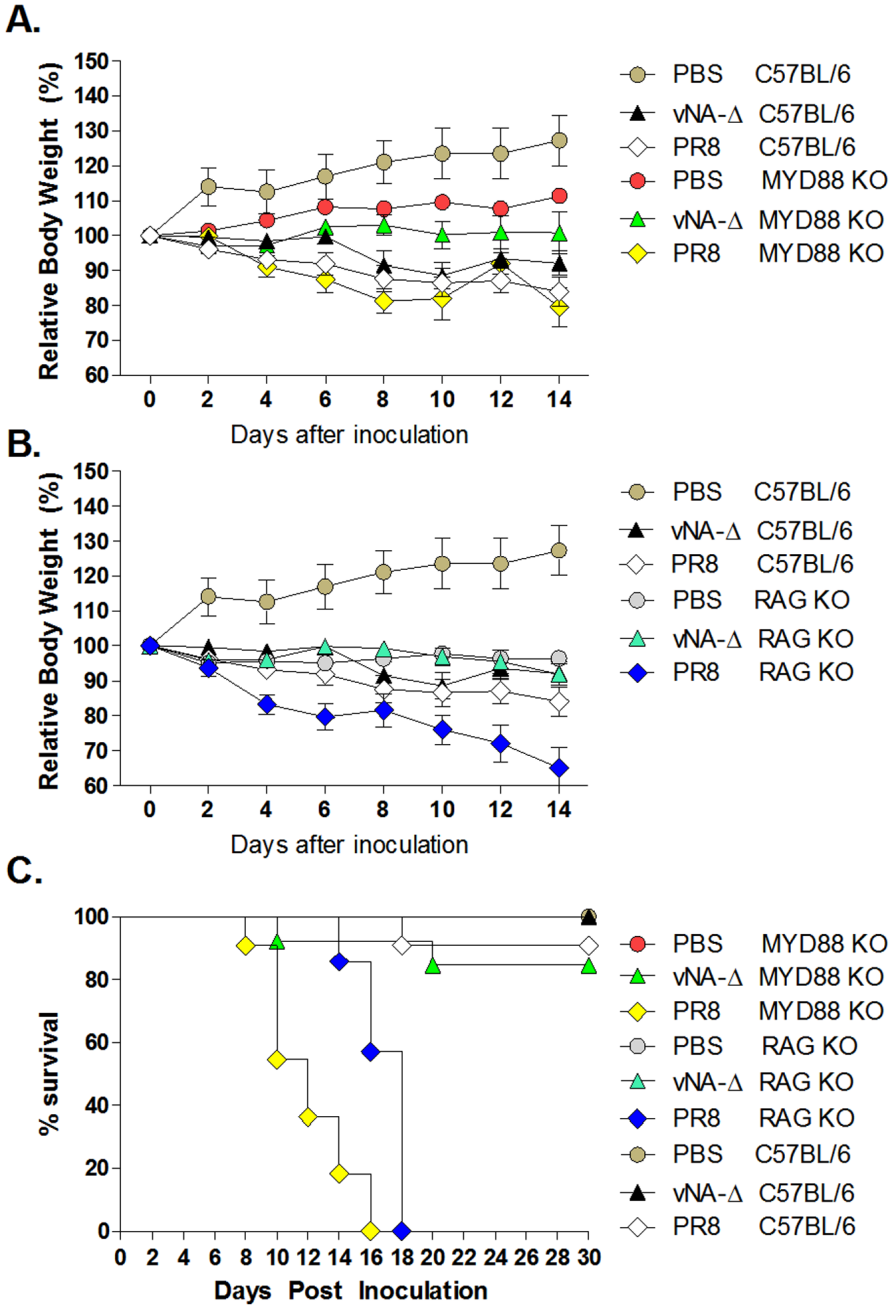
Characterization of recombinant vNA-Δ virus in immunodeficient mice. C57BL/6 mice, MyD88 -/- and RAG -/- mice were anesthetized and inoculated with PBS (mock) or 5×10^3^ PFU of the PR8 or 5×10^4^ PFU of the recombinant vNA-Δ virus. The weight loss (A and B) and mortality (C) were followed (n  =  9–12 in each group; data represents two independent experiments). Results depicted in figure A and B were obtained from the same experiment.

One drawback of the use of recombinant viruses is the potential hazard of such vectors to immunocompromised hosts [18], [19], [20]. Thus, we evaluated the virulence of recombinant vNA-Δ in mice lacking innate (MyD88 -/-) or acquired (RAG -/-) branches of immune response. To this aim, KO mice and WT C57BL/6 mice were anesthetized and inoculated with PBS (mock) or infected intranasally with a sub lethal inoculum of 5×10^3^ PFU of PR8 or with a higher inoculum of 5×10^4^ PFU of vNA-Δ. Weight loss and mortality of inoculated mice were tracked during the experiment (**figure 7**). C57BL/6 mice inoculated with PR8 virus displayed dramatic weight loss and one out of ten inoculated mice died. In addition, all the Myd88 -/- (n = 11) and RAG -/- (n = 7) mice inoculated with PR8 virus displayed abrupt weight loss (**Figure 7A and 7B**) and died (**figure 7C**). In sharp contrast, 87% of Myd88 -/- (n = 13) and 100% RAG -/- (n = 10) mice inoculated with vNA-Δ survived to the challenge (**figure 7C**). Taken together, these results suggest that recombinant viruses lacking functional neuraminidase are attenuated *in vivo*, even in mice severely handicapped in innate or acquired branches of immune response.

## Discussion

Recombinant influenza viruses have been proven to be valuable tools for vaccine development against infectious agents and tumors [21], [22], [23]. Therefore, some strategies to generate recombinant influenza viruses attenuated or defective for multiplication, such as replacing the part of the neuraminidase sequence by a foreign sequence have already been developed [2], [24]. However, questions about the lung and systemic inflammations triggered by those recombinant viruses as well as their potential virulence in immunodeficient hosts remain unclear. To better study these questions, we used eight plasmid driven reverse genetics to generate a recombinant influenza virus carrying only the first and the last 150 nucleotides of neuraminidase coding region, flanking a spacer, truncated neuraminidase and evaluated this recombinant virus in an experimental mouse model. Our results demonstrate the safety of this vector, which causes mild lung pathology in wild type mice and is attenuated in immunocompromised hosts. Furthermore, vaccination with the vNA-Δ induced robust T cell and humoral mediated immunity, protecting mice against the highly virulent PR8 virus influenza strain.

The first antiviral response in infected epithelial cells is type I and III interferons, which are of pivotal importance to control influenza infection and modulate immune response [25]. The inoculation with vNA-Δ elicited reduced and short lived production of IFN-β and undetectable production of IFN-λ in lungs of inoculated mice. Interestingly, we were able to detect both interferons in A549 cells infected with vNA-Δ. This apparently contradictory results could probably be explained by the fact that the A549 cells were infected in the presence of *V. cholera* neuraminidase in culture medium, which allowed full multiplication of vNA-Δ, whereas the infection of cells in mice lungs were abortive.

Because innate immunity plays a pivotal role in infection and inflammatory mechanisms, we evaluated parameters regarding neutrophils and monocytes in lungs of mice inoculated with vNA-Δ and wild type (PR8) virus. Neutrophils are important in killing infected cells through neutrophil extracellular traps (NET) and myeloperoxidase (MPO) activities [26], [27], [28]. However, the inflammatory mediators released by this cell type also relate to the immunopathology in experimental and natural influenza infection [29], [30]. Although monocytes play an important role in controlling viral infection by release of proinflammatory cytokines, they are also involved in tissue injuries triggered by influenza infection [18], [31], [32]. Importantly, vNA-Δ infection only induced low level of type I interferons and chemokines CXCL1/KC and CCL2/MCP-1 in epithelial cells, leading to a reduced influx of leukocytes and pulmonary injury. Reduced inflammatory infiltration in the lungs of mice inoculated with vNA-Δ could be also related to lower levels of IL-6 and TNF-α. Both cytokines have been associated with exacerbated inflammation and poor prognosis during influenza infection by allowing excessive recruitment of neutrophils and macrophages to the site of infection [33]. Nitric oxide, which is another hallmark of lung damage caused by influenza infection was absent in respiratory airways of vNA-Δ infected mice, reinforcing the mild character of inflammation triggered by this virus [34], [35].

In addition, we found augmented expression/production of pro-inflammatory cytokines such as type I IFN, IL-1β, IL-6, IFN-γ and TNF-α in airways of mice inoculated with PR8 virus. These cytokines are known to contribute to lung inflammation, injury and lethality [36], [37] and were barely detected in airways of mice inoculated with vNA-Δ. Interestingly, we have also found decreased levels of the counter-regulatory cytokines IL-4 and IL-10 in lungs of PR8 infected mice, whereas in lungs of vNA-Δ inoculated mice the levels of those cytokines were not altered or slightly increased, which may have contributed to the reduced inflammation found in lungs of mice inoculated with vNA-Δ. Consistently with lung results, inoculation with vNA-Δ did not increase the serum levels of TNF-α, IL-6, IFN-γ and CCL2/MCP-1, cytokines related to poor prognostic when their production is unbalanced [18], [19], [20], [38].

Another finding of our study was that inoculation with vNA-Δ resulted in the production of specific IgA and IgG antibodies in BALF and serum. Antibody levels and the antibody mediated hemagglutination inhibition were inoculum dependent. Moreover, the number of specific anti-NP CD8+ T cells in spleen elicited by inoculation with vNA-Δ was also found dependent on virus inoculum. This is particularly important since the CD8+ T cell response is known to play a pivotal role in controlling primary influenza infection [18], [19], [20], [38], [39].

Although vaccination with 10^3^ and 10^5^ PFU of vNA-Δ was able to protect the inoculated mice against the challenge infection with PR8 virus, only the group that received the higher vNA-Δ dose (10^5^ PFU) was completely protected. Therefore we believe that both higher levels of neutralizing antibodies and CD8+ T cells elicited by the higher dose of vNA-Δ could be an explanation for the full protection that we observed after challenge with PR8 virus. This explanation is reinforced by the recent demonstration of cooperativity among neutralizing antibodies and CD8+ T cells resulting in a robust protective immunity against influenza infection [40], [41].

Interestingly, after challenge infection we found a significantly higher number of specific anti-NP CD8+ T cells in mice vaccinated with vNA-Δ. These results could be due to the reduced amounts of IgA and IgG elicited by inoculation with vNA-Δ, which were unable to completely neutralize PR8 influenza virus during challenge, triggering a more robust cell mediated response in the lungs of those animals. Indeed, most of the animals inoculated with PR8 or 10^5^ PFU of vNA-Δ virus displayed no viral load in the lungs after challenge infection whereas all animals inoculated with 10^3^ PFU of vNA-Δ harbored virus in the lungs after challenge.

One of the most important aspects in the vaccine development field is safety in immunocompromised hosts. Thus, we also evaluated the safety of influenza virus without an enzymatically active neuraminidase in severely immunocompromised mice. vNA-Δ was attenuated in MyD88 -/- and RAG -/- mice, which are unable to trigger toll-dependent (with exception of TLR3) innate immune responses [41] and lack B and T lymphocytes, respectively [42]. Remarkably, all the RAG -/- mice inoculated with vNA-Δ survived the challenge, whereas some degree of virulence was maintained in Myd88 -/- mice since 13% died. Overall, our results suggest that although vNA-Δ would be safe for hosts with a functional adaptive immune response. Further studies should be done to better understand the role of vaccination in severely immunocompromised hosts especially those with compromised innate immunity. It is important to note that vNA-Δ elicits an abortive infection, therefore precluding the risk of vaccinated people shedding and spreading this virus.

In conclusion, we have demonstrated that vaccination with recombinant influenza viruses truncated in neuraminidase gene causes mild infection with reduced lung inflammation in wild type mice and the virus is attenuated even in severely immunocompromised mice. In addition, vNA-Δ elicited strong humoral and cellular viral immune responses, protecting vaccinated mice against challenge with a highly virulent strain of influenza virus. Hence, considering that the vNA-Δ virus expressing a heterologous protein is viable and induces a strong protection against influenza, our study gives support to the use of such recombinant influenza viruses in development of safe bivalent vaccines against influenza and other pathogens.

## Material and Methods

### Ethical Statement

This study was carried out in strict accordance with the recommendations in the Guide for the Care and Use of Laboratory Animals of the Brazilian National Council of Animal Experimentation (http://www.cobea.org.br/) and the Federal Law 11.794 (October 8, 2008). All animal studies were approved by the Ethical Commission on Animals Use (CEUA/Fiocruz, license L-001/09).

### Mice

MyD88-/-, Rag -/- mice and their respective control mice (C57BL/6) matched by sex and age (8–12 weeks old) were obtained from the animal facilities of the Federal University of Minas Gerais (Centro de Bioterismo [CEBIO], Belo Horizonte, Brazil) and Centro de Pesquisa René Rachou (CPqRR/FIOCRUZ) and were housed according to standard institutional guidelines.

In all infection procedures, animals were anesthetized and kept under observation until they completely recovered. We anesthetized mice before euthanasia procedures for all *in vivo* experiments.

For weight loss and survival measures the animals were monitored at different times after inoculation. For survival curves, the animals were anesthetized and euthanized by cervical dislocation when they reached certain degree of weight loss (25%), except for differentiation of innate and adaptative immune response, in which we used a different weight loss endpoint to better differentiate the mortality curves (35%).

### Cells

MDCK and A549 cells were grown at 37°C under 5% CO_2_ atmosphere in Dulbecco's modified Eagle Medium (DMEM; SIGMA) with 1 mM sodium pyruvate, 4.5 mg/ml L-glucose and antibiotics (100 U/ml penicillin and 100 µg/ml streptomycin: MDCK; 20 µg/ml of gentamicin and 5 µg/ml of amphoterecin B: A549), herein called complete DMEM medium and supplemented with 5% heat inactivated fetal bovine serum (FBS; CUTILAB). 293T cells were grown at 37°C under 5% CO_2_ in complete DMEM supplemented with 10% FBS.

### Plasmids for influenza reverse genetics

The pPRNA plasmid was constructed as previously described and encodes the wild type neuraminidase and segments of the A/WSN/33 (H1N1, herein named WSN) [43]. Plasmid pPRNA38 codes for a recombinant WSN NA segment where the entire NA ORF is followed by a duplicated 3' promoter, a *Xho*I/*Nhe*I linker, a duplication of the last 42 nucleotides (nt) of the NA ORF and the original 5' promoter [44]. In order to construct the truncated neuraminidase segment, PCR amplified products were generated using the pPRNA plasmid as template. These generated amplicons contained the sequence of hepatitis δ ribozyme followed by 19nt of 3′ non-coding region and the first 150nt of NA coding region. To generate the plasmid pPR150×42nt, a PCR amplification product, herein named NA150REV was cloned into the pPRNA38 vector digested with *Sac*I and *Xho*I restriction enzymes. Next, we generated another PCR product, using pPRNA as template which contained the last 150nt of the NA coding region, which were followed by the 28nt of 5′ non-coding region and the truncated human RNA polymerase I promoter. This amplicon was cloned into pPR150×42nt digested by XhoI and HindIII enzymes resulting in transfer plasmids carrying truncated NA sequence named pPRNA169×178 (**figure 1A**). All transfer plasmids were first analyzed by digestion profile using the appropriated restriction enzymes and then sequenced using Dynamic ET Dye Terminator Cycle Sequencing KIT (AMERSHAM) and a Megabace 1000 automatic sequencer (AMERSHAM). The sequence of 660 nucleotides which encodes for no protein was cloned into the plasmid pPR169×178 and digested as described above to construct the plasmid pPR169-SPC-178. Influenza A/PR8/34 bidirectional transfer plasmids pHW2000-HA, NA, M, NS, PB2, PB1, PA and NP were kindly provided by Dr. Ron Fouchier (Erasmus of Rotterdam Institute, Netherlands) [44].

### Generation of recombinant viruses

Wild type PR8 were generated by eight plasmid driven reverse genetics as described by de Wit [45]. Briefly, recombinant influenza viruses harboring a truncated NA segment and carrying the spacer sequence (herein named vNA-Δ) were generated as described by de Goede and co-workers with modifications [45], using the transfection reagent Fugene HD (ROCHE). Infectious viral particles were recovered from cell culture supernatants and they were cloned twice by limit dilution technique on MDCK cells. Viral work stocks were prepared by infecting MDCK cells cultivated in complete DMEM supplemented with 2 µg of Trypsin-TPCK, 0.3% of bovine serum (BSA) and 500 µU/ml of type III *Vibrio cholerae* neuraminidase (SIGMA). Viral stocks were titrated on MDCK cell monolayers, in standard plaque assays under agarose overlay.

### Viral RNA extraction and RT-PCR analysis

Viral RNA (vRNA) extraction from cell-free supernatants of infected MDCK cells and Reverse Transcriptase-PCR analysis were performed as previously described [10]. Amplicons were analyzed on 1% agarose gel and visualized by ethidium bromide staining. RT-PCR products were purified using QiaEXII kit (Qiagen). The presence of mutations was determined by sequencing using Dynamic ET Dye Terminator Cycle Sequencing KIT (AMERSHAM) and a Megabace 1000 automatic sequencer (AMERSHAM).

### Measurement of type I and III interferons in cell culture

In order to evaluate the induction of interferon beta (IFN-β; a type I IFN) and interferon lambda 28a (IFN-λ2; a type III IFN) genes by recombinant influenza virus, A549 cells were seeded in 6-well plates (5×10^5^ cells/well). Twenty-four hours later cells were infected with two M.O.I of PR8 or recombinant vNA-Δ or incubated with the same culture media containing BSA, Trypsin and *Vibrio cholerae* neuraminidase described above without virus (*Vc*NA treated control). At different time points, the cells were harvested and total cellular RNAs were extracted with RNeasy kit (QIAGEN), according to manufacturer recommendations and RT-PCR reactions were performed as previously described [46]. Quantitative PCRs were done using a Lightcycler Real Time PCR Machine (Applied Biosystems). Result analysis was performed using SDS 2 software. All data were normalized by the respective beta-actin levels and expressed as a ratio relative to non-infected cells (*Vc*NA treated control) cultured using the same conditions as the infected ones (*in vitro* assays) or PBS inoculated (mock) mice (*in vivo* assays). PCR primers used for human genes were previously described [47].

### Influenza challenge and immunizations

Mice (wild type, MyD88 -/- or RAG -/-) were anesthetized with 15 mg/kg of ketamine and 0.6 mg/kg of xylazine and inoculated intranasally with PBS (mock), vNA-Δ or PR8 virus in 25 µl of PBS. For survival, weight loss, lung histological and inflammatory assessments wild type mice were inoculated using 10^5^ PFU of either recombinant or PR8 virus. For challenge and acquired immune response one group prime immunized using 10^3^ PFU of recombinant vNA-Δ was included. For infection of immunocompromised mice (RAG -/- or MyD88 -/-) or wild type control (C57BL/6 mice), the animals were inoculated with either 5×10^3^ PFU of PR8 or 5×10^4^ PFU of vNA-Δ. The weight of inoculated animals was assessed at indicated time points. Survival of inoculated animals was followed over 10–30 days.

To evaluate influenza multiplication in mouse lungs, the animals were euthanized at defined time points after infection and lung homogenates were prepared in 3 ml of PBS. Viral loads in lungs were assessed by standard titration under agarose overlay on MDCK cells.

### Harvest of bronchoalveolar lavage fluid (BALF) and lung myeloperoxidase (MPO) and N-acetylglucosaminidase (NAG) measurement

At the indicated time points after infection, mice were euthanized with an overdose of ketamine/xylazine solution. Subsequently, BAL was harvested by washing the lungs twice with two 1mL aliquots of PBS [47]. After centrifugation, the pellet was used for total and differential leukocytes counts of stained slides. The supernatant (BAL) was used for cytokines, chemokines, total protein and nitrite measurements. After BALF harvesting lungs were perfused with 5 ml of PBS to remove circulating blood and frozen. A hundred mg of tissue was homogenized in PBS with anti-proteases to perform ELISA, MPO and NAG assays, as previously described [48].

### Measurement of cytokines in mice BALF, serum and tissues

At the indicated time points, PR8 virus, vNA-Δ and PBS (mock) inoculated mice were anesthetized and blood was collected from brachial plexus. After death, BALF samples were harvested as described above. The levels of the cytokines IL-6, IL-10, IL-12p70, IFN-γ, TNF-α and MCP-1 in serum were assessed by BD CBA Mouse Inflammation Kit (Becton Dickinson) according to manufacturer's instructions. The levels of CCL2, CCL11, CXCL1 and CXCL9 in BALF, and the lung levels of IL-1β, IL-4, IL-6, IL-10, IFN-γ, TNF-α were assessed by ELISA according to manufacturer's instructions (R&D systems, Minneapolis) as previously described [49]. The induction of IFN-β and muIFN-λ2/3 genes was measured by qRT-PCR, after lung tissue total RNA extraction and reverse transcription using primers specific for murine samples.

### Assessment of protein and Nitrite levels in BALF

Total protein levels were measured in BALF using the Bio-Rad Protein Assay kit, using a BSA standard curve, according to manufacturer's instructions [38]. The product of Nitric Oxide oxidation, nitrite, was measured by adding 100 µL of BALF and 100 µL of Griess reagent (1% sulfanilamide and 0.1% naphthylethylenediamide in 5% phosphoric acid) and comparing to the absorbance at 550 nm to a standard curve of sodium nitrite [50].

### Histopathological analysis

Histopathological changes induced by infection in the lungs of mice inoculated with either PR8 virus or vNA-Δ were analyzed by a pathologist blind to the experiment, using lungs of PBS inoculated mice as control. Lung left lobes were fixed in formalin and further dehydrated gradually in ethanol, embedded in paraffin and cut into 4-mm sections. Slides preparations containing the processed tissue were stained with H&E and examined under light microscopy and scored by the pathologist. The score system was performed as previously described [50]. Briefly, airway, vascular and parenchyma inflammation, neutrophilic and mononuclear infiltration and epithelial injury were assessed in 27 points of score. Photomicrography was performed using an optical microscope with a MotiCam Digital camera with a built-in 3.0 MegaPixel sensor.

### Detection of anti-influenza antibodies

Blood and BAL of mice infected with recombinant vNA-Δ or PR8 virus were collected at pre-determined time points after inoculation. Serial dilutions of serum samples were used to determine flu-specific antibodies titers by ELISA using PR8 virus as antigen. Briefly, 96-well ELISA plates (NUNC Maxisorp) were coated with 0.5 µg of detergent-disrupted purified PR8 virus per well in 0.2 M Na-carbonate buffer, pH 9.6 (overnight at 4°C). Bound antibody was detected with anti-mouse total IgG (H+L; Amersham) and IgA antibodies (SouthernBiotech) carrying the Horseradish Peroxidase and revealed by the addition of TMB peroxidase substrate (KPL) as indicated by the supplier.

Serum was used for hemagglutinin inhibition (HI) assay, and to this aim, serial dilutions (2 fold) of mice sera were incubated with 4 hemagglutinin units (HU)/25 µl of PR8 virus and 1% turkey red blood cells. HI titers were determined as the highest serum dilution able to completely inhibit hemagglutination.

### ELISPOT

Spleens of immunized mice were obtained two weeks after the inoculation with PR8 virus or vNA-Δ (10^3^ or 10^5^ PFU) or two weeks after the challenge with 10^5^ PFU of PR8. Single cell suspensions from mice spleens were prepared as previously described [51], [52]. Spleen cells were adjusted to 1 × 10^6^ cells per well in cell culture medium. For stimulation, a final concentration of 10 µg/ml of nucleoprotein (NP) of PR8 ASNENMETM peptide (NP; amino acids 366-374; for H-2K^b^) was added. ELISPOT assay were performed essentially as previously described [53]. The spots were counted on ImmunoSpot S5 Core Analyzer (CTL).

### Statistical analysis

Statistical significance for ELISA and ELISPOT assays were evaluated using Mann-Whitney test (non-parametric data). The survival distributions were analyzed by *log-rank* test. Inflammatory profile in lungs and BAL were evaluated by one-way ANOVA, with post-test Newman-Keuls. We performed the ESD method (extreme studentized deviate; or Grubbs' test), to determine whether one of the values in the list is a significant outlier from the rest. The software GraphPad Prism 5 was used to analyze data and make graphs.

## Supporting Information

Figure S1 **Inflammatory profile in BAL following infection with PR8 or vNA-Δ virus.** C57BL/6 mice were inoculated with PBS (mock) or infected intranasally with10^5^ PFU PR8 or vNA-Δ. Mice were euthanized (n  =  6–8 in each group) 1, 4 and 7 dpi and bronchoalveolar lavage was performed. Each cell type was counted (BAL) and the respective chemokine measured in BALF by ELISA. Neutrophils and CXCL1 (A), macrophages/monocytes and CCL2/MCP-1 (B), lymphocytes and MIG/CXCL9 (C), eosinophils and Eotaxin/CCL11 (D) levels were determined. Data are presented as mean ±SEM. *, ** and *** for p<0.05, p<0.01 and p<0.001, respectively, when compared to mock or indicated groups (one-way ANOVA, Newman-Keuls).
(RAR)

Figure S2 **Measurement of cytokines in BALF.** C57BL/6 mice were inoculated with PBS (mock) or infected intranasally with 10^5^ PFU of influenza PR8 or vNA-Δ. Mice were euthanized 1, 4 and 7 dpi (*n*  =  5). The levels of the cytokines IFN-γ (A), MCP-1 (B), IL-6 (C) and TNF-α (D) were assessed in BALF samples by BD CBA Mouse Inflammation Kit (Becton Dickinson) according to the manufacturer's instructions. Data were evaluated by Mann-Whitney test *, ** and *** for p<0.05, p<0.01 and p<0.001 respectively.
(RAR)

Figure S3 **Evaluation of PR8 virus titer in the lungs of vaccinated mice after challenge.** C57BL/6 mice were inoculated intranasally (25 µl of inoculum) with 10^3^ PFU of /PR8, 10^3^ or 10^5^ PFU of vNA-Δ, or PBS (mock; n  =  5–7 in each group). Twenty-one days after the prime-inoculation the animals were challenged with a lethal dose of 10^5^ PFU of PR8. Mice were euthanized 4 dpi and virus titers were quantified in the lungs. Data were evaluated by Mann-Whitney test *, ** and *** for p<0.05, p<0.01 and p<0.001 respectively.
(RAR)
